# Successful Treatment of Spontaneous Intracranial Hypotension With Subdural Hematoma With Cervical Epidural Blood Patch: A Case Report

**DOI:** 10.7759/cureus.78893

**Published:** 2025-02-12

**Authors:** Chun Peng Goh, Shi Yu Gu, Yi Xiu Chua, Ira Sun, Shiong Wen Low

**Affiliations:** 1 Neurosurgery, National University Health System, Singapore, SGP; 2 Neurosurgery, Ng Teng Fong General Hospital, Singapore, SGP; 3 Radiology, Ng Teng Fong General Hospital, Singapore, SGP; 4 National University Health Services Group, Ng Teng Fong General Hospital, Singapore, SGP

**Keywords:** epidural blood patch, orthostatic headaches, spontaneous cerebrospinal fluid leak, spontaneous intracranial hypotension (sih), subdural hematomas

## Abstract

Spontaneous intracranial hypotension (SIH) is characterized by orthostatic headache. In severe cases, SIH can be complicated by symptomatic subdural hematoma. There is a lack of consistency and established treatment guidelines for the management of SIH. Lumbar epidural patch is the recommended first-line invasive treatment after failing conservative management.

We present the case of a 42-year-old man with SIH complicated by symptomatic left subdural hematoma, which was successfully treated with surgical drainages and targeted cervical epidural blood patch.

The primary aim of management of SIH is to stop cerebrospinal fluid leakage, and if the necessary specialty is available, targeted cervical epidural blood patch may be proposed as first-line invasive treatment. Symptomatic subdural hematoma should be managed with prompt action such as surgical drainage or middle meningeal artery embolization.

## Introduction

Spontaneous intracranial hypotension (SIH) is characterized by orthostatic headache of spontaneous onset that is due to low cerebrospinal fluid (CSF) pressure or a CSF leak and is usually accompanied by nuchal pain, tinnitus, auditory disturbances, phonophobia, and nausea [1]. Initially described by Georg Schaltenbrand in 1938 [2], SIH is now understood to arise from a reduction in CSF volume caused by spontaneous CSF leaks from the thecal sac rather than solely from decreased CSF pressure [3]. The resulting reduction in CSF volume induces brain sagging within the cranial vault, placing traction on the sensory nerves of the meninges and bridging veins, which clinically manifests as headache. These headaches are exacerbated in an upright position due to increased traction on the meninges. Additionally, compensatory vasodilation of cerebral vessels in response to low CSF pressure exacerbates the headache by increasing brain volume [4].

The incidence of SIH is estimated to be 5 per 100,000 annually, with a peak occurrence around the age of 40. Females are affected twice as often as males [5]. CSF leaks responsible for SIH typically arise from dural defects, which may be congenital or acquired, often secondary to trauma. Contributing factors include structural defects in the dura surrounding nerve root sheaths, osteophytic protrusions, congenital connective tissue disorders, intervertebral disc herniation, and the presence of meningeal diverticula [6].

Indicative magnetic resonance imaging (MRI) features of low CSF pressure include brain sagging, tonsillar descent, posterior fossa crowding, dilated epidural veins, diffuse pachymeningeal enhancement, and subdural collections containing either blood or CSF [7]. When MRI results are normal or inconclusive, diagnostic invasive techniques such as radioisotope cisternography, CT myelography, or digital subtraction myelography may effectively identify the precise site of the leak [8].

Subdural hematoma (SDH) is a significant complication associated with SIH. Although the exact pathophysiology underlying SDH in SIH patients is not fully understood, several mechanisms have been proposed. One hypothesis suggests that the downward displacement of the brain, resulting from reduced CSF pressure, causes tearing of the bridging veins within the dural border cell layer, leading to their rupture. Alternatively, the gradual enlargement of subdural CSF collections may increase tension on the bridging veins, predisposing them to rupture in certain cases [9]. If left untreated, the presence and progressive enlargement of an SDH can result in significant mass effect, leading to severe neurological symptoms and potentially life-threatening complications.

We present a case of a young man with SIH complicated by bilateral SDH, which was successfully treated with three surgical drainages and targeted cervical epidural blood patch.

## Case presentation

A 42-year-old male presented to the emergency department twice with neck pain radiating to the occipital region. He has no significant past medical history and was not on any regular medications. The MRI of the cervical spine showed mild degenerative changes, and symptoms were attributed to cervicogenic headache, which improved after analgesics.

**Figure 1:**
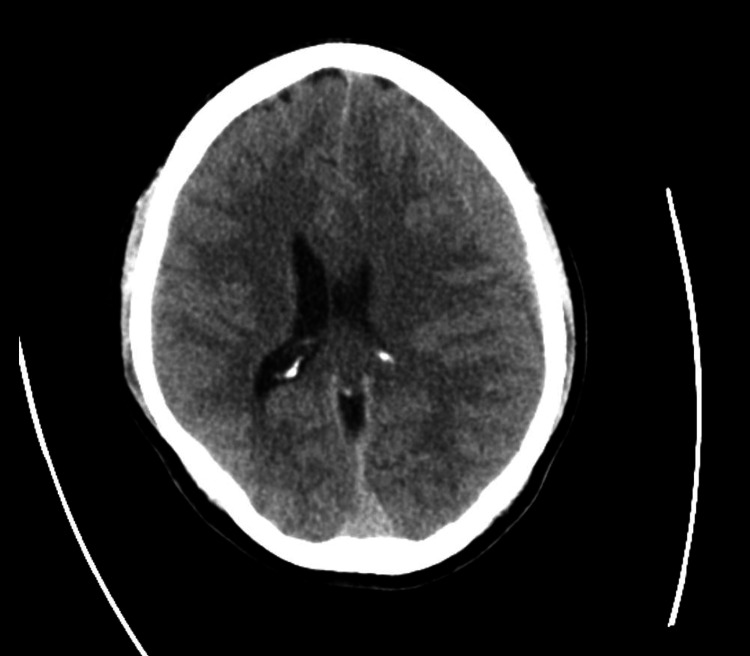
First CT of the brain showing bilateral chronic SDH, measuring 1.5 cm in thickness on the left and 0.5 cm on the right.

**Figure 2:**
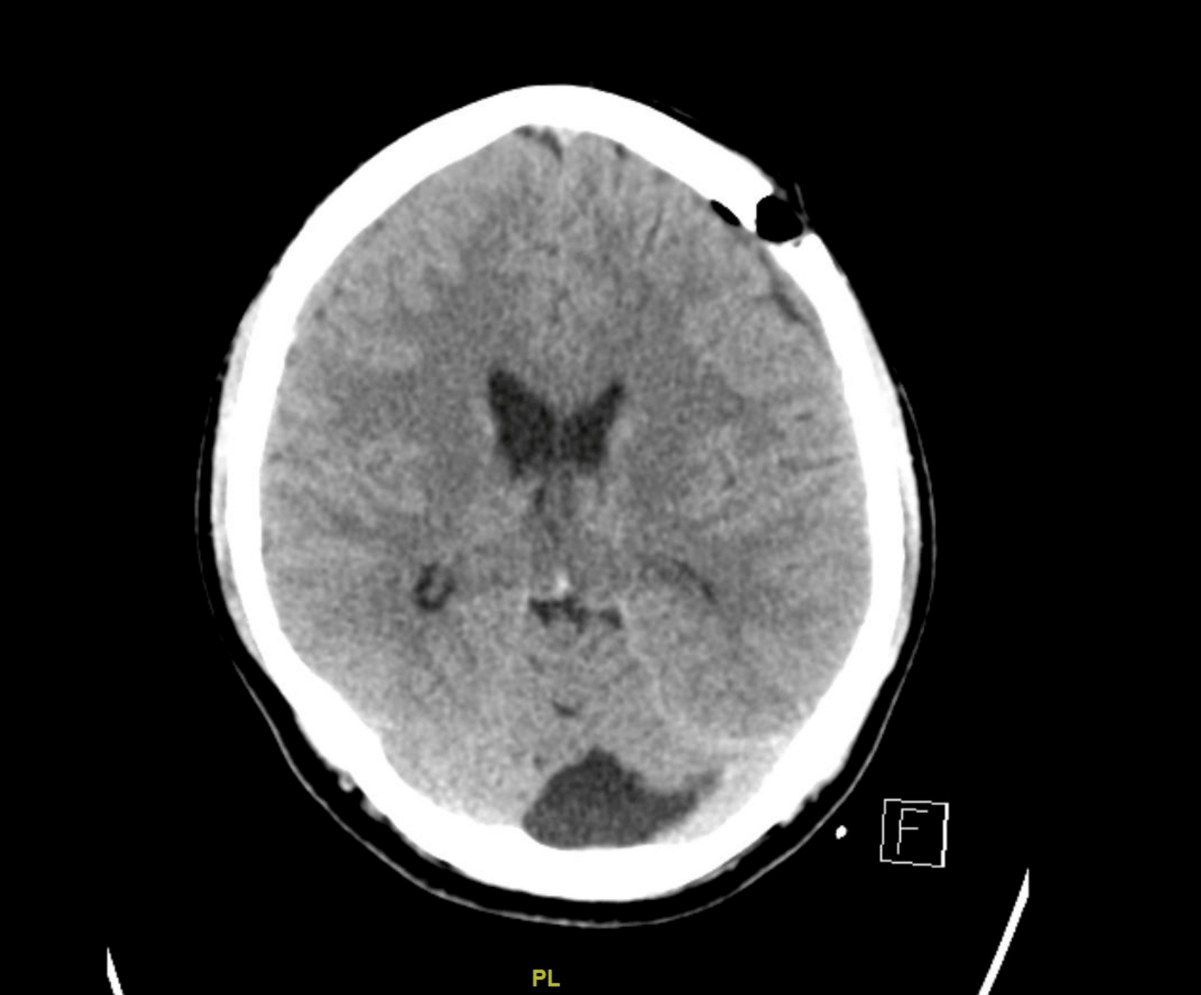
CT of the brain after burr hole drainage of the left SDH showing improvement of mass effect.

Three months later, he was brought to the emergency department with lethargy, headache, vomiting, and generalized weakness. His GCS on arrival was E3V4M6. CT of the brain revealed bilateral chronic SDH, measuring 1.5 cm in thickness on the left and 0.5 cm on the right (Figure 1). Given the lack of history of head injury, CT angiogram was performed, which showed no vascular abnormalities. He underwent burr hole drainage of the left SDH, which improved his symptoms (Figure 2), and was discharged two days later.

**Figure 3:**
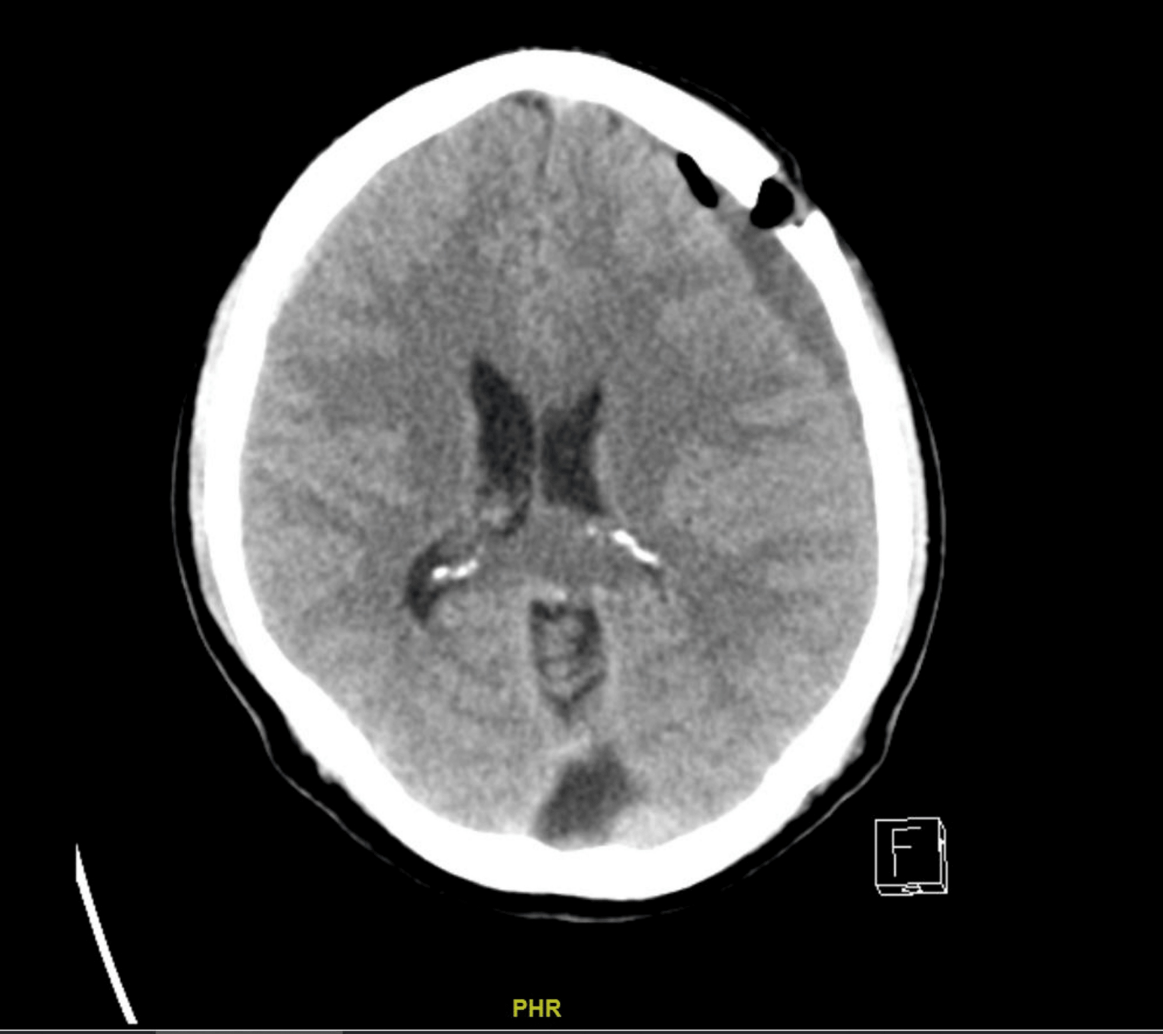
Recurrence of the left subdural hematoma with midline shift.

He was re-admitted three days after discharge with worsening malaise. A repeat CT of the brain confirmed re-accumulation of the left SDH (Figure 3) and underwent re-do drainage of the left SDH. He again experienced good recovery.

**Figure 4:**
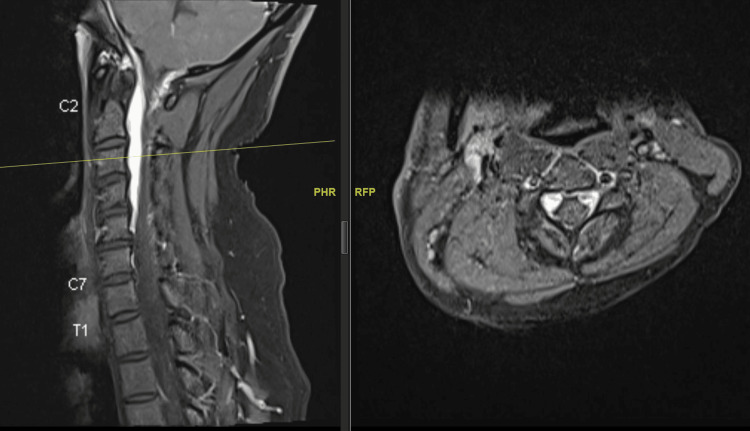
T1-contrasted images showing engorged epidural veins in the upper cervical spine.

**Figure 5:**
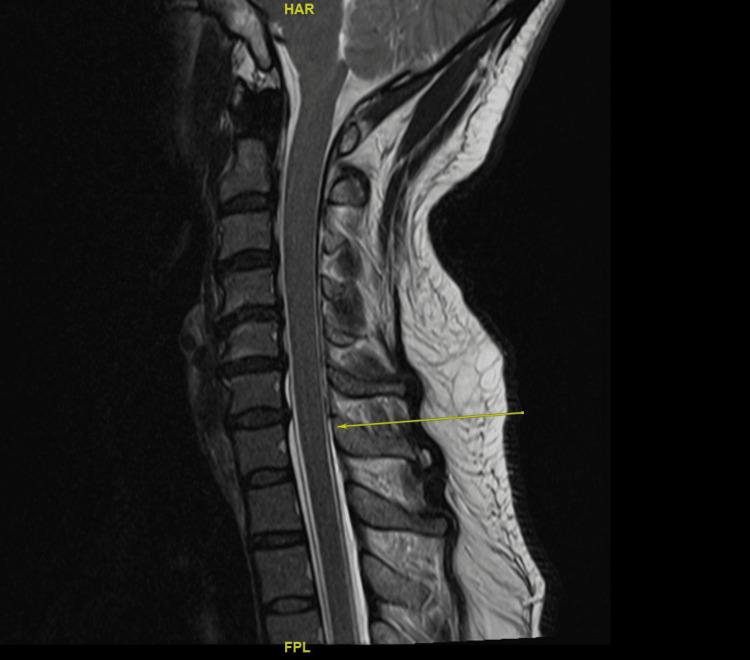
T2-weighted image showing epidural fluid collection extending from C6 down (yellow arrow).

A contrast-enhanced MRI of the brain showed the presence of residual bilateral SDH as well as evidence of intracranial hypotension, including dural enhancement and venous engorgement in the craniocervical junction. Dedicated MRI of the cervical, thoracic, and lumbar spine was performed, which showed engorged epidural veins in the upper cervical spine (Figure 4) and the presence of epidural fluid collection extending from C6 down to the lumbar region (Figure 5). The exact location of the leak could not be identified on MRI after discussion with a neuroradiologist but was likely to be from around C6 given that most of the CSF collection was seen here.

**Figure 6:**
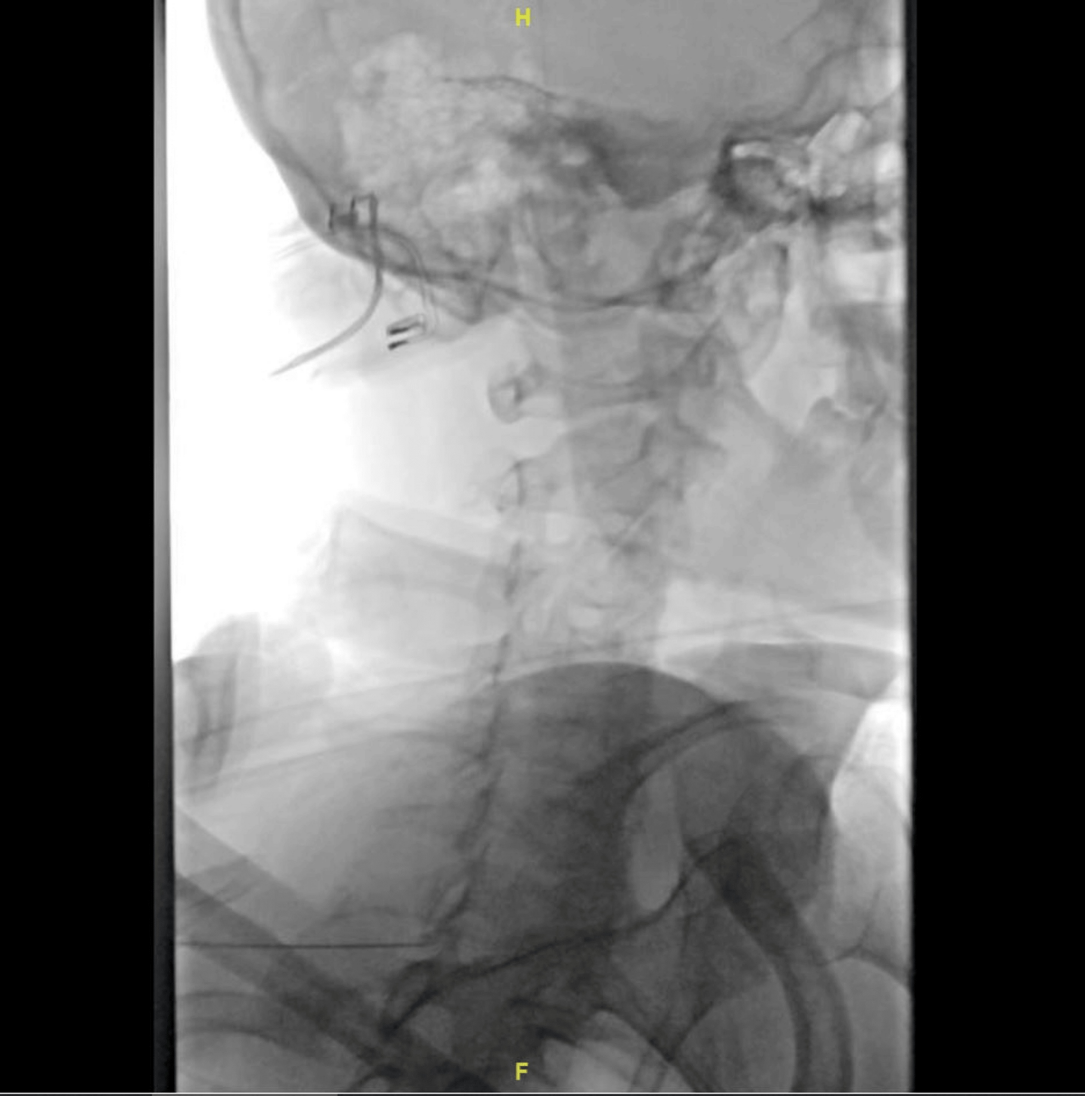
Cervical epidural blood patch performed at the C7/T1 level under fluoroscopic and CT guidance.

**Figure 7:**
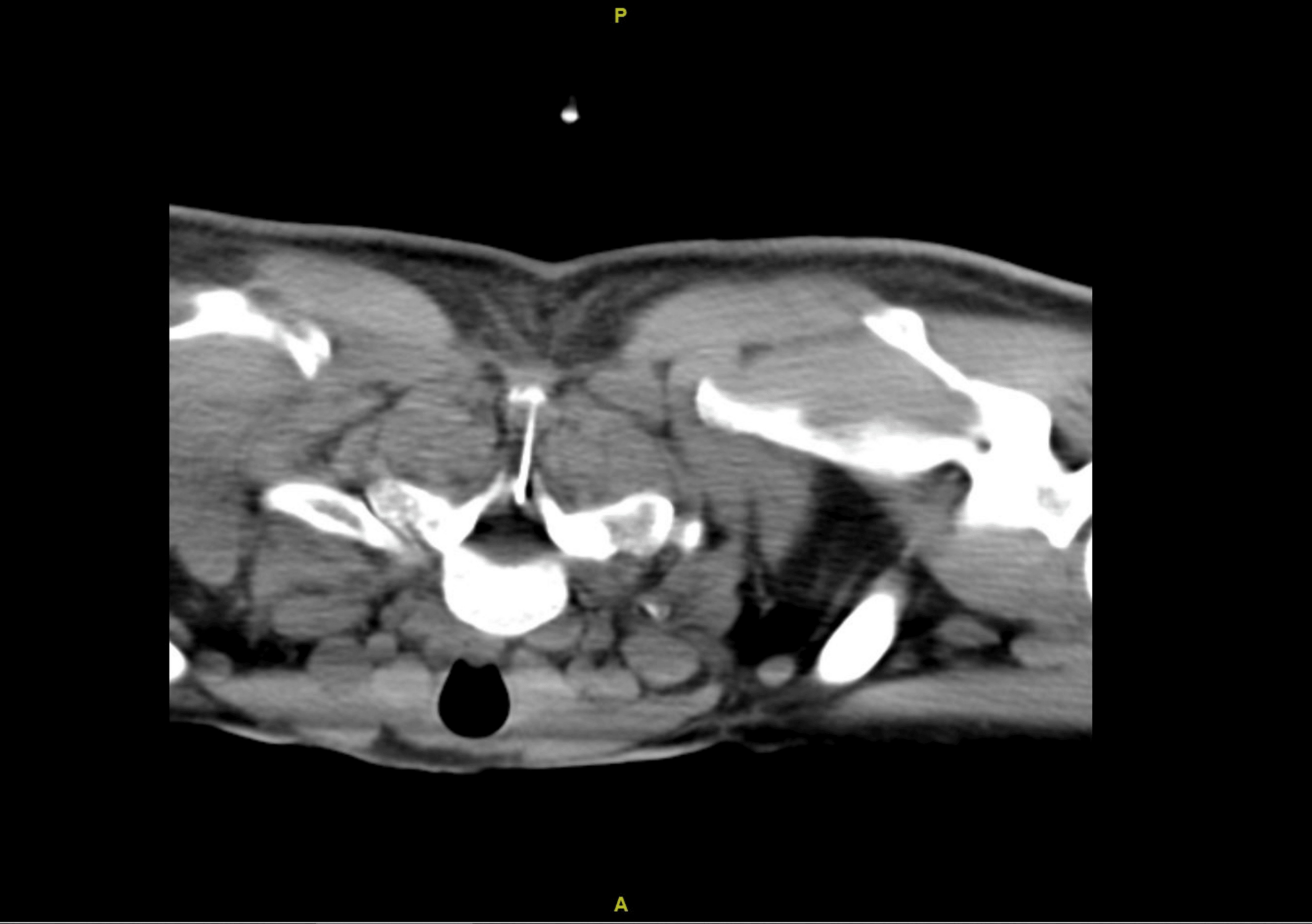
Position of the spinal needle confirmed with CT.

**Figure 8:**
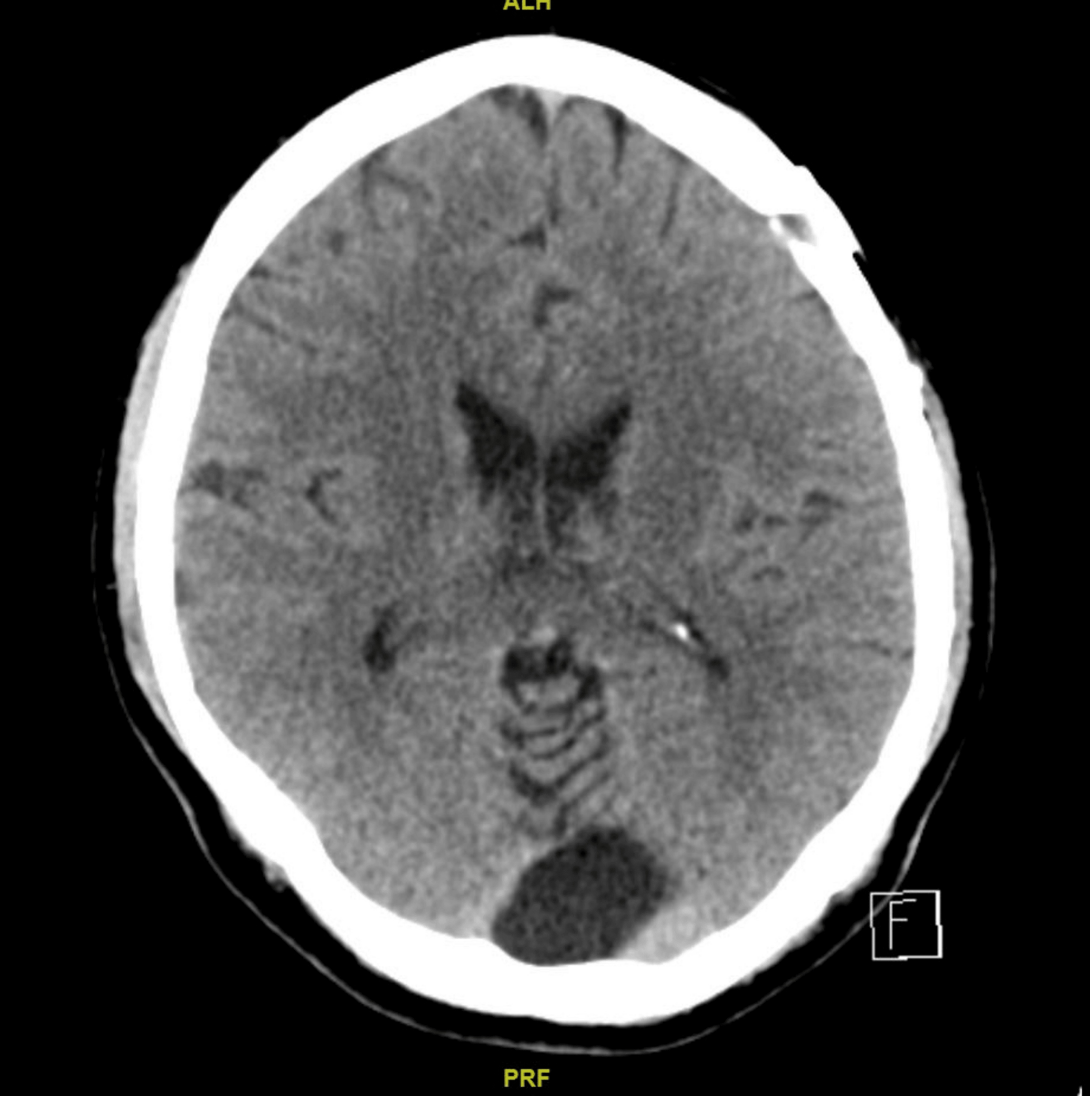
CT of the brain nine months after discharge showing complete resolution of bilateral subdural hematoma.

Decision was made for a cervical epidural blood patch at the C7/T1 level, which was chosen due to safety reasons with a cuff of epidural fluid at this level. This was performed by a consultant interventional radiologist under fluoroscopic (Figure 6) and CT guidance (Figure 7). A 21G spinal needle was guided into C7/T1 posterior midline epidural space, and satisfactory position was confirmed with CT. This was followed by an injection of 10 mL of the patient's own blood to the epidural space. His neck pain and symptoms related to SIH had resolved. CT of the brain at nine months (Figure 8) showed complete resolution of SDH. He returned to work with no noticeable cognitive deficit.

## Discussion

Patients with SIH typically present with orthostatic headaches, often accompanied by nausea, vomiting, neck stiffness, visual disturbances, and a range of other neurological symptoms [10]. If untreated, the orthostatic component of the headache may diminish or disappear over time. Pain can be diffuse or localized and is rarely unilateral [3]. Misdiagnosis is common due to a lack of familiarity with the disease process [1].

Indeed, our patient initially presented with neck pain and occipital headache that worsened towards the end of the day. His symptoms were initially attributed to a cervicogenic headache, which was managed with analgesics. He was admitted through the emergency department three months later with symptomatic bilateral chronic SDHs.

In 2023, consensus guidelines on the management of SIH were proposed by Cheema et al. [11]. Initial treatment for mild-to-moderate SIH-related headaches includes bed rest, hydration, caffeine, and abdominal binders for up to two weeks.

For invasive treatment, large-volume non-targeted lumbar epidural blood patches are recommended as first-line treatment. If the patient remains symptomatic, a repeat epidural blood patch should be considered after five days [11]. Autologous blood should be administered until the patient reports symptoms such as back pain, pressure, headaches, or radicular symptoms, with a maximum volume of 40 mL and an optimal minimum total volume of 20 mL [11].

If a CSF leak is identified in the cervical region, targeted epidural blood patches should be considered for patients who remain symptomatic despite appropriate conservative management and/or a non-targeted epidural blood patch [12]. A targeted blood patch at the site of the leak is preferred over placement at other levels [13], as Beards' study suggests that the blood clot predominantly extends only three to five segments from the injection site [14]. Studies have shown that blood injected at the lumbar level does reach the cervical levels; however, the amount of blood needed to form a stable clot is unclear [15].

Cervical epidural blood patch is associated with risks, including spinal cord compression, cranial nerve palsies [16], altered mentation, SDHs, seizures [17], and transient bradycardia. However, in a literature review by Kapoor and Ahmed including 19 patients, neck pain or pressure was identified as the most common complication, with no observed neurological or vascular complications [13].

Techniques such as direct epidural blood patch under CT [18] and O-arm guidance [19] and utilizing an epidural catheter to reach the dural leak site have been described [10]. The epidural space narrows from the lumbar to the cervical region, and the required blood volume is determined by the onset of pain or radiculopathy. Most studies used blood volumes between 5 to 8 mL (range: 2.5 mL to 15 mL) at the cervical level [10,13,18]. Our patient underwent a CT-guided injection of 10 mL of blood at the cervical leak site.

Surgical repair is recommended when the location of the CSF leak has been precisely identified, and epidural blood patches have proven ineffective. The procedure should be performed by a neurosurgeon with expertise in spinal CSF leaks. The choice of surgical technique will depend on the specific characteristics of the leak. In cases where a CSF-venous fistula is identified through myelography, endovascular treatment may be considered as a first-line option [20].

The presence of symptomatic SDHs in this case necessitated emergency surgical drainage to release the SDH. However, as the etiology of the SDH is not addressed, the probability of recurrence is high. In our case, the patient experienced a recurrence of SDH three days after the initial burr hole drainage, which resulted in another surgery.

A recent case report suggested that embolization of the middle meningeal artery (MMA) could be an effective approach to address the issue [12]. MMA embolization has been demonstrated to disrupt the blood supply to the SDH and may offer a less invasive alternative to surgical drainage [21].

## Conclusions

In managing patients with SIH, the primary aim is to stop CSF leakage. If the necessary specialty is available, targeted cervical epidural blood patch may be proposed as first-line invasive treatment. If SIH is complicated by symptomatic SDH, prompt action such as surgical drainage or MMA embolization, is necessary to reverse symptoms related to mass effect on the brain.

## References

[1] (2018). Headache Classification Committee of the International Headache Society (IHS) The International Classification of Headache Disorders, 3rd edition. Cephalalgia.

[2] Schaltenbrand G (1938). Neuere Anschauungen zur Pathophysiologie der Liquorzirkulation [New observations on the pathological physiology of the cerebrospinal fluid circulation]. Zentralbl Neurochir.

[3] Limaye K, Samant R, Lee RW (2016). Spontaneous intracranial hypotension: diagnosis to management. Acta Neurol Belg.

[4] Mokri B (2001). The Monro-Kellie hypothesis: applications in CSF volume depletion. Neurology.

[5] Deline C, Schievink WI, Cedars-Sinai Cedars-Sinai (2025). Spontaneous Intracranial Hypotension. https://rarediseases.org/rare-diseases/spontaneous-intracranial-hypotension/.

[6] Schievink WI, Jacques L (2003). Recurrent spontaneous spinal cerebrospinal fluid leak associated with "nude nerve root" syndrome: case report. Neurosurgery.

[7] Sun-Edelstein C, Lay CL, Tung GA (2020). Spontaneous intracranial hy­potension: pathophysiology, clinical features, and diagnosis. UpToDate.

[8] Schievink WI (2006). Spontaneous spinal cerebrospinal fluid leaks and intracranial hypotension. JAMA.

[9] Kim BW, Jung YJ, Kim MS, Choi BY (2011). Chronic subdural hematoma after spontaneous intracranial hypotension : a case treated with epidural blood patch on c1-2. J Korean Neurosurg Soc.

[10] Kwon SY, Kim YS, Han SM (2014). Spontaneous C1-2 cerebrospinal fluid leak treated with a targeted cervical epidural blood patch using a cervical epidural Racz catheter. Pain Physician.

[11] Cheema S, Anderson J, Angus-Leppan H (2023). Multidisciplinary consensus guideline for the diagnosis and management of spontaneous intracranial hypotension. J Neurol Neurosurg Psychiatry.

[12] Cirillo L, Verna F, Princiotta C (2024). Spontaneous intracranial hypotension and subdural hematomas treatment management using MMA embolization and target blood patch: a case report. Life (Basel).

[13] Kapoor SG, Ahmed S (2015). Cervical epidural blood patch--a literature review. Pain Med.

[14] Beards SC, Jackson A, Griffiths AG, Horsman EL (1993). Magnetic resonance imaging of extradural blood patches: appearances from 30 min to 18 h. Br J Anaesth.

[15] Ferrante E, Arpino I, Citterio A (2006). Is it a rational choice to treat with lumbar epidural blood patch headache caused by spontaneous cervical CSF leak?. Cephalalgia.

[16] Perez M, Olmos M, Garrido FJ (1993). Facial nerve paralysis after epidural blood patch. Reg Anesth.

[17] Kardash K, Morrow F, Béïque F (2002). Seizures after epidural blood patch with undiagnosed subdural hematoma. Reg Anesth Pain Med.

[18] Rai A, Rosen C, Carpenter J, Miele V (2005). Epidural blood patch at C2: diagnosis and treatment of spontaneous intracranial hypotension. AJNR Am J Neuroradiol.

[19] Takai K, Taniguchi M (2017). Targeted epidural blood patch under O-arm-guided stereotactic navigation in patients with intracranial hypotension associated with a spinal cerebrospinal fluid leak and ventral dural defect. World Neurosurg.

[20] Borg N, Oushy S, Savastano L, Brinjikji W (2022). Transvenous embolization of a cerebrospinal fluid-venous fistula for the treatment of spontaneous intracranial hypotension. J Neurointerv Surg.

[21] Désir LL, D'Amico R, Link T (2021). Middle meningeal artery embolization and the treatment of a chronic subdural hematoma. Cureus.

